# Phosphopeptide interactions with BRCA1 BRCT domains: More than just a motif

**DOI:** 10.1016/j.pbiomolbio.2015.02.003

**Published:** 2015-03

**Authors:** Qian Wu, Harry Jubb, Tom L. Blundell

**Affiliations:** Department of Biochemistry, 80 Tennis Court Road, University of Cambridge, CB2 1GA, Cambridge, United Kingdom

**Keywords:** BRCT, Tandem BRCT domains, BRCA1, Phosphopeptide, DNA damage response, pSXXF motif, DDR, DNA damage response, PIKKs, phosphoinositide 3-kinase-related kinases, *BRCA1*, breast cancer susceptibility gene 1, HBOC, hereditary breast and ovarian cancer, BRCT, BRCA1-C-Terminal, PPII, polyproline II

## Abstract

BRCA1 BRCT domains function as phosphoprotein-binding modules for recognition of the phosphorylated protein-sequence motif pSXXF. While the motif interaction interface provides strong anchor points for binding, protein regions outside the motif have recently been found to be important for binding affinity. In this review, we compare the available structural data for BRCA1 BRCT domains in complex with phosphopeptides in order to gain a more complete understanding of the interaction between phosphopeptides and BRCA1-BRCT domains.

## 1 Introduction: from BRCT domain to the BRCA1 tandem BRCT domains

Maintaining the integrity of genetic information is key to the survival of cells. Eukaryotes have evolved to host sophisticated cell-cycle-dependent regulation networks to deal with DNA damage arising from both exogenous and endogenous sources. Dynamic protein–protein interactions and protein complex assemblies in the signalling cascades mediating DNA damage response (DDR) are often initiated by protein post-translational modification such as phosphorylation. Reversible interaction interfaces are created by phosphorylation on serine/threonine residues by key DDR-signalling-cascade regulators that include phosphoinositide 3-kinase-related kinases (PIKKs) (ATM, ATR, DNA-PK) (Matsuoka et al., 2007, Meek et al., 2008) and checkpoint-effector kinases (Chk1, Chk2 and MK2) (Reinhardt and Yaffe, 2009).

Deficiency in DDR regulation networks caused by gene mutation could potentially lead to cell death or tumorigenesis. Mutations in the tumour suppressor gene *BRCA1* (breast cancer susceptibility gene 1) were first identified in patients with hereditary breast and ovarian cancer (HBOC) (Futreal et al., 1994, Hall et al., 1990, Miki et al., 1994). Further analysis of this 1863-amino-acid BRCA1 protein led to the identification of the BRCT (named as **BR**CA1-**C**-**T**erminal) domain (Koonin et al., 1996). Even though BRCT domains share low sequence identities (about 14%), they have been successfully identified in many proteins involved in RNA processing, cell checkpoint regulation and DNA damage response and repair (Bork et al., 1997, Callebaut and Mornon, 1997, Woods et al., 2012), where BRCT domains mediate interactions with proteins, DNA (Yamane and Tsuruo, 1999, Yamane et al., 2000) and poly(ADP-ribose) (PAR) (Pleschke et al., 2000).

**Fig. 1:**
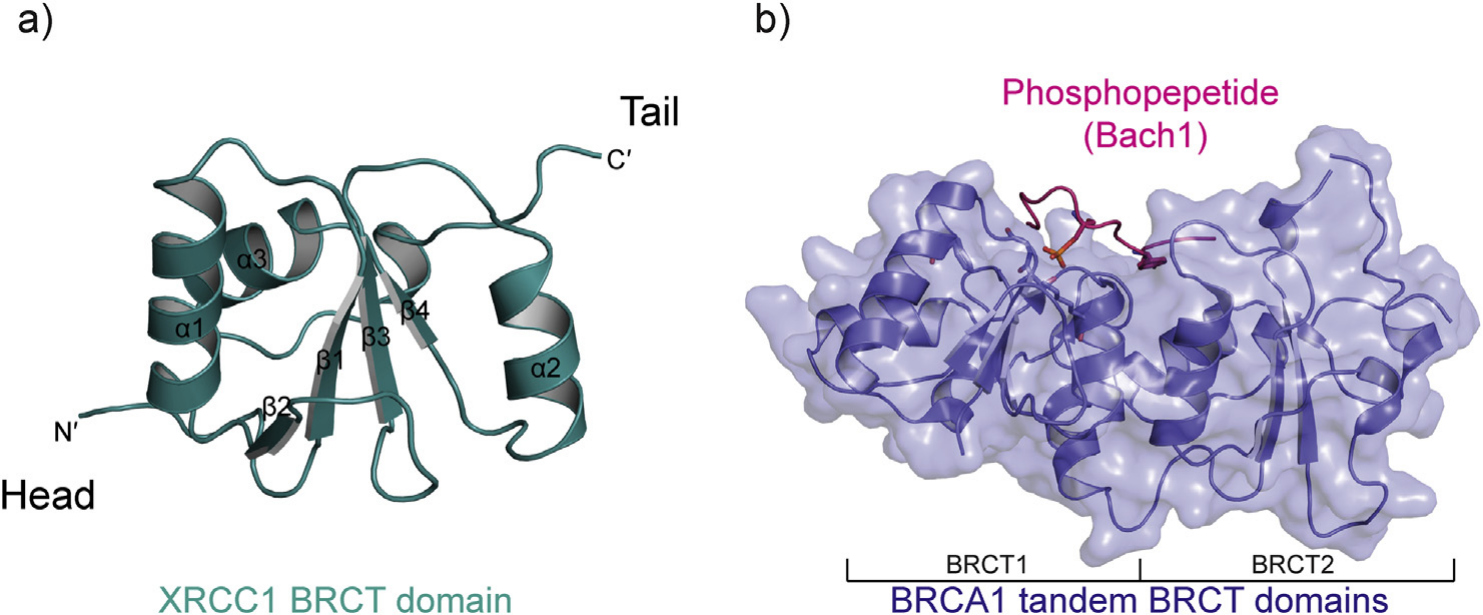
BRCT domain structures. a) Crystal structure of the second BRCT domain of XRCC1 (PDB: 1CDZ) (Zhang et al., 1998). The ribbon representation of the mainchain is in dark green. N′ (Head) and C′ (Tail) ends are labelled. b) Crystal structure of BRCA1-BRCT tandem 1 and 2 domains in complex with Bach1 phosphopeptide (PDB: 1T29) (Shiozaki et al., 2004). Bach1 phosphopeptide binds to the cleft between the two BRCT domains. The ribbon representation of BRCT domain mainchain is in slate colour and the globular structure is represented by a transparent surface. Bach1 phosphopeptide is in pink.

A typical BRCT domain comprises between 90 and 100 amino-acid residues and folds as a globular domain with secondary structural elements ordered as βαββαβα. The first crystal structure of a BRCT domain, that of XRCC1, defined a four-stranded parallel β-sheet surrounded by three α-helices, in which helices α1 and α3 locate on one side of the β-sheet and α2 on the other side (PDB: 1CDZ) (Zhang et al., 1998) (Fig. 1a). 23 human genes have been identified encoding BRCT domain-containing proteins and 12 of these contain more than one BRCT domain within the sequence (Mesquita et al., 2010, Woods et al., 2012), as does BRCA1 where the two BRCT domains are packed tightly in tandem. One of the most significant properties for BRCA1 BRCT domains is their ability to bind phosphorylated proteins containing the sequence motif pSXXF (where p indicates phosphorylation) (Manke et al., 2003, Rodriguez et al., 2003, Yu et al., 2003) (Fig. 1b). Potential phospho-independent interaction has also been reported recently for interaction of BRCA1-BRCT domains with DNA-PKcs (Davis et al., 2014). Together with the N-terminal RING domain, C-terminal BRCT domains are the sites of the main BRCA1 mutations found in patients with breast and ovarian cancer (Couch and Weber, 1996, Friedman et al., 1994, Shattuck-Eidens et al., 1995). Studies of mouse models have shown that BRCT-domain phosphoprotein binding but not the RING-domain E3-ligase activity is required for Brca1 tumour suppression (Shakya et al., 2011). Comprehensive reviews of the evolution and function of BRCT domains can be found in Leung and Glover, 2011, Mesquita et al., 2010. Here, we focus on the interface between the phosphopeptide and BRCA1 BRCT domains.

## 2 2D-structure comparisons of BRCA1 tandem BRCT domains bound with phosphopeptides

**Fig. 2:**
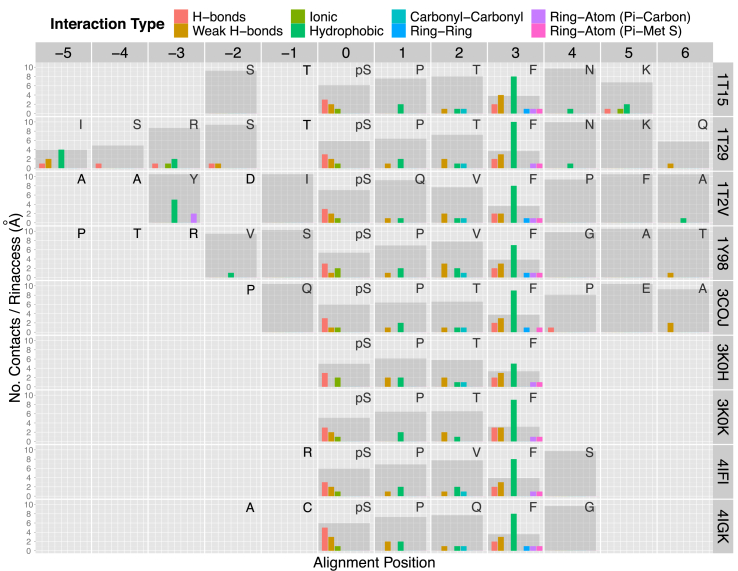
2D-interaction map of phosphopeptide with BRCA1-tandem-BRCT domains. Phosphopeptides in BRCA1-bound conformations from X-ray crystal structures (PDB codes on right hand side) were aligned by sequence (only residues in positions from −5 to +6 are shown) and their interaction profiles with BRCA1 determined. Coloured bars indicate the number of interactions of a given type formed between the peptide and BRCA1 at each residue position (numbered by position in alignment, top). Binding depth in Ångstroms, measured by Rinaccess as the smallest spherical probe size that can fit into a surface cavity (Kawabata, 2010), is shown as larger, translucent grey bars. Smaller Rinaccess values indicate deeper binding depth of the residue into BRCA1. Structures of complexes include BRCA1 BRCT bound with Bach1 (PDB: 1T15 and 1T29) (Clapperton et al., 2004, Shiozaki et al., 2004), motif tetrapeptides (PDB: 3K0K and 3K0H) (Campbell et al., 2010), AcetylcoA carboxylase 1 peptide (PDB:3COJ) (Shen and Tong, 2008), CtIP (PDB: 1Y98) (Varma et al., 2005), ATRIP (PDB: 4IGK) (Liu and Ladias, 2013), optimized peptide (PDB:1T2V) (Williams et al., 2004) and BAAT1 (PDB:4IFI) (Liu and Ladias, 2013).

The availability of protein structure data for BRCA1 tandem BRCT domains alone and in complex with various phosphopeptides has enabled us to understand and compare the protein-peptide interaction in detail. We first describe these interactions using a “2D interaction map” method, built upon the structural interaction fingerprint methodology used in the CREDO structural interactomics database (Schreyer and Blundell, 2013) (Fig. 2). All bound phosphopeptides identified in protein structures were sequence aligned, with the phosphorylated Ser residue, which mediates key H-bonds in the pSXXF motif, placed at position 0. The side chain of the Phe at the +3 position inserts into a deep hydrophobic binding pocket, forms hydrogen-bond interactions via its mainchain atoms, and interacts with the sulphur in BRCT M1775 via a ring π interaction. Outside the pSXXF motif region, the N- and C-terminal peptides also contribute to the interaction. The structure of Bach1 in complex with a phosphopeptide (PDB: 1T29) (Shiozaki et al., 2004) defines an especially large interface outside the motif. The optimized phosphopeptide in the BRCA1 BRCT-phosphopeptide complex (PDB:1T2V) (Williams et al., 2004) has a Tyr at position −3 in the N-terminal region, which forms hydrophobic and ring-atom interactions through its side chain. The “2D interaction map” in Fig. 2 provides a clear visualization for comparing phosphopeptide interactions with BRCT domains as well as for defining the depth of the binding pockets.

## 3 The “two anchors” binding in pSXXF motif region

The 3D structures of BRCA1-tandem-BRCT domains show that the two BRCT domains (BRCT1 and BRCT2) are associated in a head-to-tail manner (Williams et al., 2001). A large hydrophobic interface between BRCT1 and BRCT2 is created by α2 (from BRCT1) and α′1 and α′3 (from BRCT2), with an extra linking helix αL between the two domains, next to the α′3 of BRCT2. Using the BRCA1-Bach1 structure (PDB: 1T29) (Shiozaki et al., 2004) as an example, Bach1 phosphopeptide can be seen to bind to the cleft generated between two tightly packed BRCT domains (Fig. 1b). The pSXXF motif in Bach1 phosphopeptide functions as a “two-anchor” interaction mode (also described as “two-knobs” in Shiozaki et al. (2004))) with the BRCT domain, in which the phosphorylated Ser (position 0) and the Phe (position +3) interact with the BRCT1 and BRCT2 individually (Fig. 2b). Comparison with a BRCT-domains-only structure (PDB: 1JNX) (Williams et al., 2001) by structural superposition and alignment with the BRCT1-domain structure shows that the BRCT2 domain moves closer to BRCT1 when bound to a phosphopeptide (Fig. 2b). A larger degree of phosphopeptide-induced domain movement was also observed in TopBP1 BRCT 7/8- Bach1 complex (Leung et al., 2010). This suggests that phosphopeptide binding “tightens” the structure of tandem BRCT domains.

### 3.1 The first and second “anchors”

**Fig. 3:**
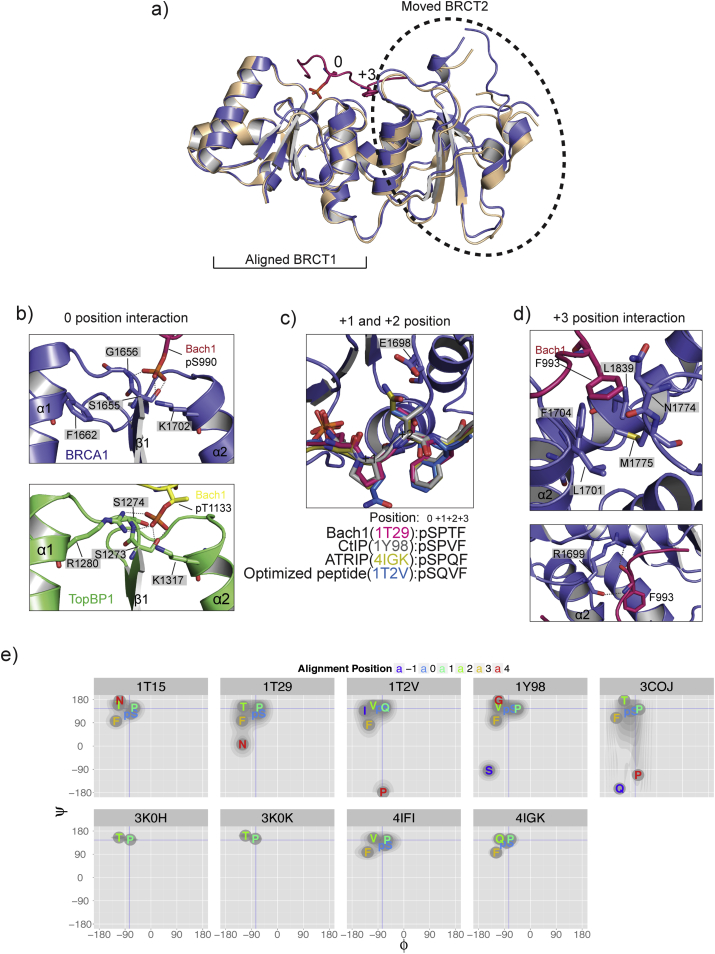
BRCA1-tandem-BRCT-domain interaction with phosphopeptide. a) Aligning the BRCT1 domain for structures of Bach1-phosphopeptide-bound BRCT domains (PDB: 1T29) (Shiozaki et al., 2004) and BRCT domains alone (PDB:1JNX) (Williams et al., 2001) demonstrates the movement of the BRCT2 domain. b) 0 position phosphate group interaction with BRCT domains from BRCA1 (PDB: 1T29) (Shiozaki et al., 2004) and Topbp1 (PDB: 3Al3) (Leung et al., 2010). c) +1 and +2 positions of phosphopeptide from Bach1 (PDB: 1T29) (Shiozaki et al., 2004), CtIP (PDB: 1Y98) (Varma et al., 2005), ATRIP (PDB: 4IGK) (Liu and Ladias, 2013) and optimized peptide (PDB:1T2V) (Williams et al., 2004) when bound with BRCA1 BRCT domains. d) +3 Phe interaction with BRCT2 domain in Bach1 (PDB: 1T29) (Shiozaki et al., 2004). In a), b), c), and d) BRCA1 BRCT domains are in slate colour, Bach1 phosphopeptide in 1T29 is in pink, TopBP1 BRCT 7-8 BRCT domains are in green, Bach1 phosphopeptide in 3AL3 is in yellow, BRCA1-BRCT-only structure is in wheat. Polar interaction is indicated in grey dashed lines. Residues labelled for the BRCT domain is highlighted with grey. e) Ramachandran plots (Ramachandran et al., 1963) of the phosphopeptides (−1 to +4 positions) in BRCT domains bound conformations. Residues are coloured by their positions in the sequence-based alignment of the pSXXF motif peptides. Blue horizontal and vertical lines intersect at the ideal phi/psi angles of proline in a polyproline II helix.

For the first “anchor”, the phosphate group in pS (0 position) mediates polar interactions with evolutionarily conserved residues S1655/G1656 in β1 and K1702 in α2 of BRCA1 (Fig. 3b Top). The phosphate group in pS cannot be replaced by the phosphate-mimicking residue glutamic acid for motif containing tetrapeptide. For example, the tetrapeptide EPTF failed to bind to BRCT domains, whereas pSPTF can (Lokesh et al., 2007). Topbp1 tandem BRCT domains (domain 7 and 8, PDB: 3AL3) recognises a phosphorylated Thr instead of Ser of Bach1 at a different site (Leung et al., 2010). The phosphate group from pT mediates an extra interaction with the R1280 residue in α1 of Topbp1 BRCT7 (Fig. 3b bottom). At the equivalent α1 site, BRCA1 contains an exposed F1662, which is consistently buried in crystal packing. For the second “anchor” interaction, the Phe side chain at +3 position inserts into a deep hydrophobic pocket in the BRCT2 domain created by L1701, F1704, N1774, M1775 and L1839. R1699 also interacts with the mainchain of Phe via hydrogen bonds (Fig. 3d). Missense mutations of interaction-key residues G1656, M1775 and R1699 can be found in breast cancer patients and cause reduced/lost phosphopeptide binding property (Cantor et al., 2001, Coquelle et al., 2011, Manke et al., 2003, Shiozaki et al., 2004, Williams et al., 2004, Yu et al., 2003). Both *in vitro* and *in vivo* assays have shown that M1775R mutant failed to bind to the BRCT domains (Clapperton et al., 2004, Williams et al., 2004). Comparing the structures of the phosphopeptide-bound BRCT domains with BRCT (M1775R) only has shown that this mutation causes the disruption of hydrophobic pocket, and therefore causes the side chain of +3 position Phe to fail to insert (Clapperton et al., 2004, Williams and Glover, 2003, Williams et al., 2004). The M1775R mutation also changed the residue preference at +3 position of phosphopeptide to acidic residues (Clapperton et al., 2004).

## 4 The X residues in pSXXF motif

Apart from the conserved “two-anchor” interaction sites, affinity differences among phosphopeptides towards BRCA1 BRCT domains can arise from the variation of the X residues at positions +1 and +2 in the pSXXF motif. Superposition of four peptides (PDBs: 1T29, 1Y98, 4IGK and 1T2V) bound to the BRCT domains shows the variation of residues at positions +1 and +2 (Fig. 3c). The surface cavity of BRCT domains in these two positions is shallower than that for positions 0 and +3, as shown in Fig. 2. Combining structural information and biophysical affinity measurements from peptides shows that the majority of phosphopeptides contain Pro at the +1 position (Lokesh et al., 2007, Yuan et al., 2011a). In order to have the sidechains of pS (position 0) and F (position +3) facing to the same side for BRCT interaction, Pro provides important restraints on the conformations of peptides towards those adopted when bound to BRCT domains, so reducing the entropic penalty of peptide binding. Interestingly, Ramachandran plots for the phosphopeptides in BRCT tandem-domains-bound conformations show that the phi/psi angles of pSXXF motif residues resemble those of the idealised polyproline II (PPII) helix, which has three residues per turn (Adzhubei and Sternberg, 1993) (Fig. 3e) and brings sidechains of positions 0 and +3 to the same side of the helix.

Residues Gln, Thr and Val are found at the +2 position (Fig. 3c). As expected for an extended helix, mutation of the +2 position from Thr to Ala reduces the phosphopeptide-binding affinity (Lokesh et al., 2007). Double mutations of residues +1 to Asp and +2 to Glu have been shown to abolish the phosphopeptide interaction with BRCT domains (Liu and Ladias, 2013). At position +2, the X residue cannot be large or negatively charged due to unfavourable interactions with residue E1698 (Liu and Ladias, 2013).

## 5 Binding outside the motif

The N- and C-terminal sequences of the peptide outside the motif also contribute towards the peptide interaction. Phosphopeptides with the same core DpSPVF sequence and peptide length, but different N-terminal and C-terminal sequences, bind to BRCA1 BRCT domains with dramatically different affinities (Liu and Ladias, 2013). In all structures available so far (as seen in Fig. 2), residues that are C-terminal to the motif contribute fewer interactions than the N-terminal residues. When there is no extension of the C-terminal sequence outside the motif, the presence of the +3 carboxylate terminus is important for binding to BRCT domains through an additional salt bridge with R1699 (Campbell et al., 2010, Yuan et al., 2011b). This extra interaction is likely present in another BRCA1-BRCT-binding protein, Abraxas/CCDC98 (Liu et al., 2007, Wang et al., 2007), which also has a C-terminal Phe at Position +3 in the pSXXF motif.

**Fig. 4:**
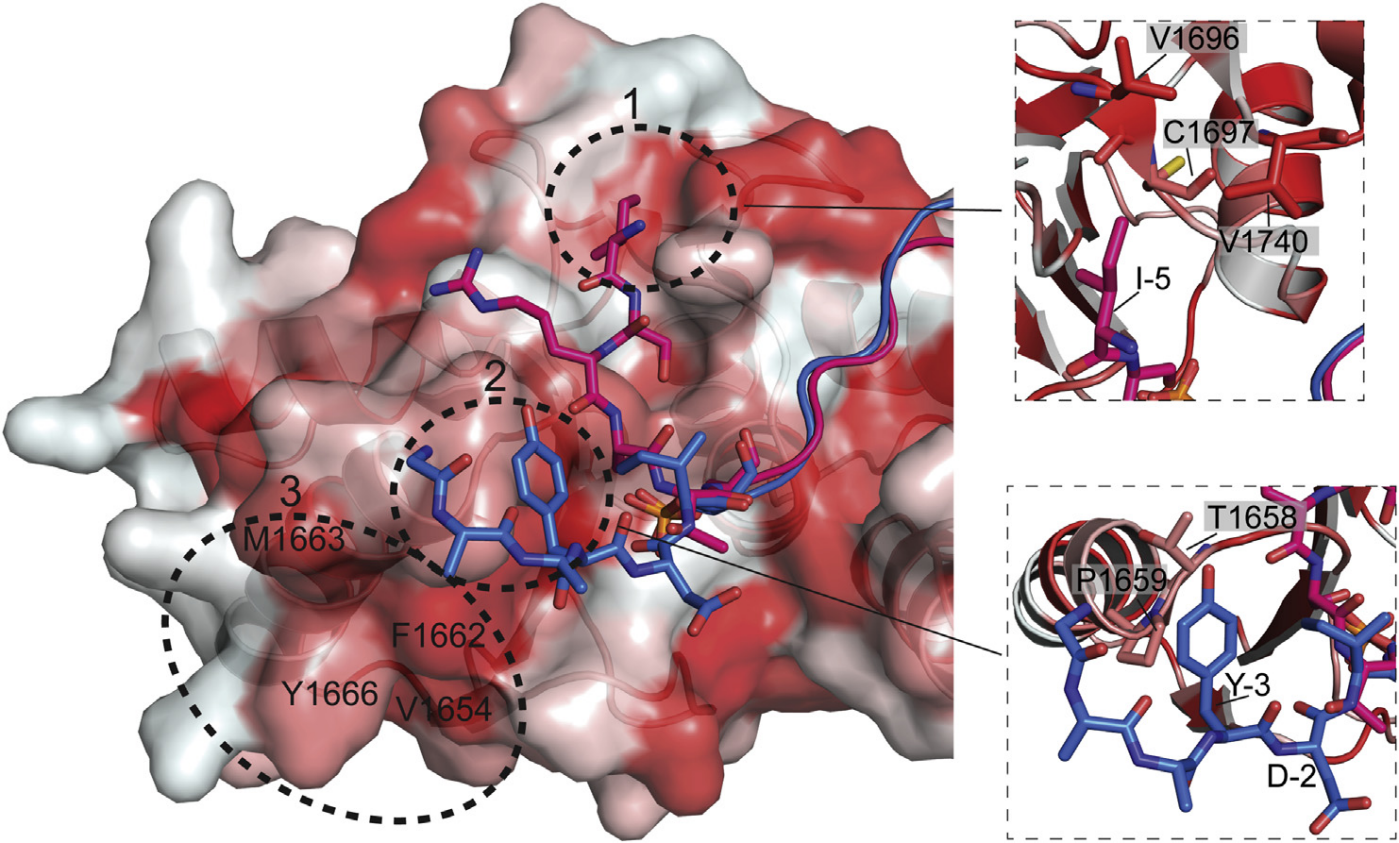
Hydrophobic regions in BRCA1-BRCT domains bound with phosphopeptide N-terminal sequence outside the pSXXF motif. The hydrophobic regions of the BRCA1-BRCT domains, indicated by red colour, comprise three patches, which are highlighted using dashed circular line. The interactions in Patches 1 and 2 are detailed on the right. Bach1 phosphopeptide from PDB: 1T29 (Shiozaki et al., 2004) is in pink, while the optimized peptide from PDB:1T2V (Williams et al., 2004) is in blue. Hydrophobic residues in Patch 3 are labelled as V1654, F1662, M1663 and Y1666.

The N-terminal region of the phosphopeptide contains more hydrophobic interactions with BRCT compared to the C-terminal sequence (Fig. 2). Surface hydrophobicity of the BRCT domains using a normalized consensus hydrophobicity scale (Eisenberg et al., 1984) is shown in Fig. 4. Residue −5 Ile in the Bach1 phosphopeptide of 1T29 structure interacts with hydrophobic Patch 1, which is formed by V1696, C1697 and V1740 in the loop region close to the interface between two BRCTs (Shiozaki et al., 2004). Patch 2, near the beginning of α1 in BRCT1, is formed by L1657 and P1659. The optimized peptide in structure 1T2V contains a Tyr at −3 position that interacts with P1659, while the negatively-charged side chain of Asp at the −2 position faces away from the BRCT domains (Williams et al., 2004). Patch 3 contains F1662, M1663 and Y1666 in α1 and V1654 in β1. This third hydrophobic patch forms isologous interactions with another molecule in the crystal for most BRCA1-BRCTs structures solved so far. It has been shown that having a residue with a naphthyl side chain at the N-terminal of the motif can increase peptide binding of the BRCT domains (Lokesh et al., 2007). The naphthyl interaction is likely mediated through hydrophobic Patches 2 and 3.

Aside from the sequence differences, phosphorylation variants in the N-terminal phosphopeptide sequence can also occur. In addition to the phosphorylation at the Ser residue in pSXXF motif, an extra Ser residue in the N-terminal region can be phosphorylated for Bach1 and Abraxas (Kettenbach et al., 2011, Wang et al., 2007). The structural and functional consequences of double phosphorylation are still unknown.

## 6 Conclusion

Initial analyses of phosphopeptide binding to the BRCA1-BRCT domains focused on the pSXXF motif (Glover et al., 2004, Leung and Glover, 2011). As more and more proteins are shown to bind to BRCA1 through the pSXXF motif, the question arises as to how these proteins compete with each other or are regulated with respect to binding to BRCA1. The variation of residues outside the canonical motif and also the residues in the “X positions” of the motif are clearly keys for control of the binding affinity and selectivity.

Our current understanding of the similarities and differences of structural interactions among these phosphopeptides bound to BRCA1-BRCT domains with respect to affinity underlines the fact that the phosphopeptides used for binding-affinity assays and structure determination usually represent only small parts of the protein structure. It is clear that we need to ask whether the rest of the phosphoprotein also contributes towards the binding with BRCA1. Understanding these detailed structures and binding modes of BRCA1 BRCT domains with various phosphopeptides can help us to understand the function of BRCA1 in various pathways and also contribute towards knowledge-based design and discovery of higher affinity BRCA1-BRCT inhibitors in the future.
